# Textronic Sensors of Hazardous Gaseous Substances

**DOI:** 10.3390/ma18020341

**Published:** 2025-01-14

**Authors:** Ewa Skrzetuska, Paulina Rzeźniczak, Zuzanna Błaszkowska, Hubert Ciszek, Olga Kowalczyk, Michał Olecki

**Affiliations:** 1 Faculty of Material Technologies and Textile Design, Textile Institute, Lodz University of Technology, 116. Żeromskiego Str., 90-924 Lodz, Poland; ewa.skrzetuska@p.lodz.pl; 2 Faculty of Electrical, Electronic, Computer and Control Engineering, Lodz University of Technology, 18. Stefanowskiego Str., 90-924 Lodz, Poland; 240853@edu.p.lodz.pl (Z.B.); 240859@edu.p.lodz.pl (H.C.); 240875@edu.p.lodz.pl (O.K.); 240890@edu.p.lodz.pl (M.O.)

**Keywords:** intelligent textiles, gas sensors, screen printing, embroidery, metal thread, acetone, methanol, toluene

## Abstract

Toxic materials are a threat in workplaces and the environment, as well as households. In them, gaseous substances are included, especially ones without any colour or fragrance, due to their non-detectability with the human senses. In this article, an attempt was made to find a solution for its detection in various conditions with the use of intelligent textiles. The approach was to perform modification on fifteen materials by screen printing using carbon nanotubes paste with expanded graphite and embroidery with stainless steel thread and then investigate their reaction with risky gases such as acetone, methanol and toluene. Four combinations of samples were tested: before tests, after the washing test and after the alkaline and acidic sweat contact test. Three materials can be highlighted. Para-aramid knitwear which reacted well to all tested gases. The biggest value of sensory percentage response was 144%. Screen-printed linen knitwear showed properly detecting skills after washing test for toluene. The biggest value of sensory percentage response was noted at 186%. The third most promising material was low surface mass cotton knitwear with embroidery which had a visible response at every stage of testing for acetone. The biggest value of sensory percentage response was 94% and the smallest one was 27%. For these three materials, repeated contact with harmful gases was tested. Simulations showed also repeated responses expressed in changes in surface resistance under changed conditions. After analysis, there is a possibility to create textile sensors for the detection of hazardous substances.

## 1. Introduction

Current times require the creation of products that not only have functional features for a given application, but are also visually attractive modern, providing the user not only with an enriched product but an enriched gadget. This fashion has begun to enter not only clothing, but also areas such as hygiene, medicine and safety. The area that connects these and many other fields has become textronic. Its goal is to create systems based on textile materials with electronic circuits. The idea is to produce intelligent textile products that support and diversify humans’ everyday life. Examples include a wide range of applications such as sparkly dresses [1], systems for monitoring the safety of employees or children in machine and car seats [2], or systems support for high-risk professions [3]. Textronic solutions can facilitate the work of not only the user, but also the person taking care of them in the case of use in medicine [4,5,6]. These solutions collect signals from the patient’s body, which can be analysed by a third party like a medical doctor. Popular ones that can be used in hospitals may be intelligent wound dressings [7,8,9] and intelligent diapers [10,11,12]. Nurses working in hospitals often have many patients under their care. These solutions can significantly improve their work.

Taking care of human health, it is important to direct attention to the issue of poisoning with harmful gases. According to the statistics published in an article by the Polish Government, during one month of the heating season in 2022, firefighters recorded 51 interventions related to carbon monoxide emissions in residential buildings. As a result of carbon monoxide poisoning, 7 people lost their lives and 22 were poisoned. Since the beginning of the heating season this year, 589 interventions have also been recorded, as many as 23 people lost their lives and 184 were poisoned [13]. Surveys conducted this year also show that only one in five households has a smoke detector installed. Accidents related to poisoning with harmful gases do not occur only in households. Chemical wholesalers and companies dealing with chemical substances are also exposed to similar incidents. According to regulations and standards, buildings must be equipped with gas sensors and the employer must provide the employee with appropriate protection. However, unpredictable situations do occur. Scientists around the world are working to strengthen this pillar in terms of awareness and improved safety.

Researchers from the Lodz University of Technology led by I. Krucińska and B. Surma patented the textile sensor detecting liquids and their fumes. Its principle of operation consists of changing resistance in the presence of the substance. The sensor is based on nonwoven fabric made from polymer micro- and nanofibers containing carbon nanotubes by the technology of forming nonwovens directly from polymer solutions (containing 3–6% carbon nanotubes by volume) [14].

Researchers from California wrote a review of flexible graphene-based wearable gas and chemical sensors. In the review, there were sensors which detect various hazardous gases as NO_2_, NH_3_, H_2_, H_2_S, CO_2_, SO_2_. Also, there were sensors detecting toxic heavy metal ions (Cd, Hg, Pb, Cr, Fe, Ni, Co, Cu, Ag) and volatile organic compounds as nitrobenzene, toluene, acetone, formaldehyde, amines, phenols, bisphenol A, and others [15].

Researchers from Ecole Polytechnique Fédérale de Lausanne and the University of Tübingen led by Danick Briand conducted a review of the works about making environmental sensors on plastic foil. They mainly focused on the temperature, humidity, and gas sensors integrated with plastic substrates [16].

Sensors of risky gases were also produced with a combination of electrospinning technology and an in situ polymerization process. The researchers from China created a yarn, which was fabricated from core-sheath polyaniline/polyacrylonitrile nanofibers. This material offered a great area for free diffusion of NH_3_ and conditions to transfer electric signal and due to that allowed the production of a textronic gas sensor detecting ammonia in exposure 10–2000 ppm, characterised by excellent repeatability and stability. Importantly, PANI/PAN nanofibers having their own conductive properties, could be used as yarns, as well as in fabric form. They also have potential in application as wearable devices because of their flexibility and mechanical properties, which make it possible to be processed into textiles by knitting, braiding, and embroidery techniques [17].

To preserve the mechanical properties of textiles, instead of modifying their composition, there is also an option to coat them with special layers. Koreans’ scientists, who produced nitric oxide-sensitive sensors, presented this approach. Its elementary component was cottoning yarn, coated with some graphene oxide and molybdenum disulfide layers. Sheets were placed on yarn with the use of Bovine Serum Albumin (BSA), which functioned as biological glue. In effect, researchers obtain an ultrasensitive NO_2_ sensor, showing a 28% response to 0.45 ppm concentration of gas in the air. Moreover, textiles are reliable even after 100 repetitive laundry tests, which makes them promise for wearable electronics applications [18].

In 2018, Indian scientists from Bharathi University developed an Ammonia sensor using a spray-coating method consisting of polyaniline-anchored MWCNTs on fabric. Thanks to polyaniline, sensors can give reliable output at room temperature, which is extremely important considering its predicted use in textiles and clothing. Results proved it to be highly selective and flexible. The sensor’s flexibility allows it to obtain stable resistance measures while bending at 150–210 degree angles. During simulations with different gases, the response to ammonia at 100 ppm was 80% while responses to gases, such as acetone, methanol, and toluene, were below 20%. This shows possibilities to explore paths to develop sensors selective to either one of those gases [19].

Another approach to detecting toxic gases has been proposed by scientists from Bandung University. The carbon monoxide sensor is based on nanocomposite graphene and ZnO zinc oxide settled on cotton material. Similar to the previous study, the method used was also deep coating. Resistance decreases during exposition to carbon monoxide. Sensor’s advantages are working at room temperature, high sensitivity and selectivity. By combining ZnO and graphene, scientists were able to obtain better responses no matter what the ratio of the composite. The addition of zinc oxide provides better selectivity. The only disadvantage is the sensor’s reaction to humidity; as it increases, the response decreases [20].

Graphene was also used for developing NO_2_ sensors by scientists in South Korea. They used reduced graphene oxide because of its high conductivity, chemical and thermal stability, and overall lightness. Another advantage in terms of textronic is mechanical properties. Sensors were made by electrospinning nylon-6 to nanofibers. It shows high selectivity to nitrogen dioxide compared to ammonia, benzene and acetone. Satisfactory results were obtained showing high durability for 5000 repeated bending tests [21].

Considering previous developments in gas textile sensors, we see a possible path of research in exploring embroidery and screen-printing methods as well as their possibilities of detecting gases such as acetone, toluene, and methanol. The aim of the research was to check the behaviour of various materials in the presence of poisonous gases, such as acetone, methanol, and toluene. Having a knowledge of their behaviour gives possibilities for making, for example, special uniforms for firefighters which would help to detect toxic gases to prevent them from causing any harm or to make special sensors which could be used on everyday items such as curtains. This article will describe the study of changes in the surface resistance of textile chemical sensors embroidered with metal threads and printed with ink paste with carbon structures under the influence of vapours of harmful substances, such as acetone, methanol and toluene. These systems can be used in home conditions, beauty salons, or chemical wholesalers.

The sensors created during the research described in this article respond to a chemical stimulus because of the adhesion of appropriate gas molecules to the surface. Depending on the tested gas, modification and base material, this process proceeded with greater or lesser intensity. In addition, due to the increased humidity in the chamber caused by the introduction of gas, the fibres of the base materials swell, which also affects the detection properties.

The novelty of the conducted work was a very wide selection of textile materials on which research was carried out related to the application of electrically conductive structures based on carbon nanotubes and expanded graphite and yarns containing stainless steel. Sensory properties for polar protic and aprotic gases and nonpolar gases were analysed. An especially important contribution to the science of wearable smart products is the work related to the durability of the developed systems in everyday use conditions, such as sweating processes, and their impact on sensor degradation and washing processes. The authors showed that textile substrates have an impact on the durability of the obtained sensors and that the types of sensor development techniques have an impact on sensitivity.

## 2. Materials and Methods

In this study, a range of 15 fabrics were selected for evaluation of their acetone, methanol and toluene detection. They consisted of natural materials, such as linen and cotton, and synthetic materials, such as viscose, polyester, and polyamide, as well as special use fabrics designed for firefighters. Selected materials are commonly used in areas such as chemical factories, beauty salons, clothing or high-risk professions. The resulting sensors will mainly seek their application in these areas to support factory workers, high-risk workers and household members exposed to the presence of the presented gases. The list of used materials is in Table 1.

Samples with dimensions 32 cm × 32 cm were cut from each of the 15 previously selected materials. Then, on the half of samples, electroconductive paths were screen printed on the samples using atrament paste with carbon nanotube and graphene, and the other metal thread with silver embroidery was embroidered on machine embroidery. The embroideries were made on a Tajima Temx-C1201embroidery machine (Nagoya, Japan) using dedicated software (Pulse Tajima-KJ05038-20.11.2013).

**Figure 1:**
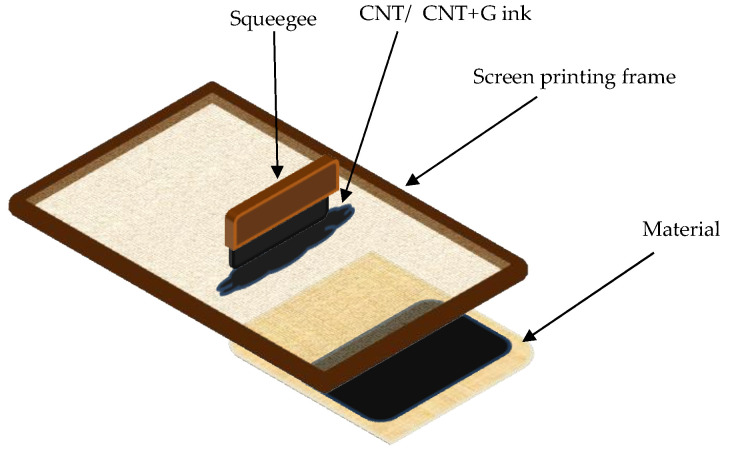
Schematic of screen printing method [22].

The paste consisted of an aqueous dispersion of carbon nanotubes from Nanocyl, under the name “Aquacyl AQ0301”, containing 3% carbon nanotubes enriched with 3% of Nanografi’s highly conductive Micron expanded graphite powder. Expanded graphite powder is composed of natural flake graphite through a chemical or electro-chemical treatment process. Expanded graphite has all the outstanding characteristics of natural crystalline graphite with high-temperature resistance, corrosion resistance, and self-lubricity. The paste composition is as follows: D_50_ (µm): 20, Powder Density (g, I): 120, Carbon Content (% at 800°C; 20 h) > 96, and Moisture Content (at 110 °C; 8 h) < 5%. The printing process consisted of 2 steps. Firstly, the sample was stretched onto a screen-printing sieve to provide the best coverage of ink during the second step–printing. The ink was pressed through a sieve using a squeegee–blunt tool used to provide better integration of ink and the weave of the fabric (Figure 1).

The process of each method is the same for each material. In the case of screen printing, the critical point is the bonding of the paste with the base material which is why 15 materials were tested to select those with the best conductive properties, i.e., those with which the paste bonded most permanently. In the case of machine embroidery, one embroidery pattern was embroidered. The execution of the embroidery also depended on the base thickness, surface mass or type of weave. The samples had to be left for 24 h for the ink paste to completely dry and bond permanently with the base material. With thread made of a blend of stainless steel, an electroconductive path, measuring around 5 cm long and 3 mm wide, was embroidered onto every fabric. The equipment used for this purpose was a Tajima embroidery machine with dedicated software. The embroidery was made in 100% filling. Both embroidery and screen-printing were then cut into smaller samples with dimensions appropriate to each of the utility tests described below. In each study, 10 samples were tested.

Surface mass measurements were conducted following the PN-EN 12127:2000 standard [23]. Each type of fabric was sampled with a 10 cm^2^ area, and then weighed using a PS 750/X laboratory scale from Radwag (Radom, Poland).

The washing procedure followed A1S conditions with steel balls, using ECE PHOSPHATE REFERENCE DETERGENT B as the washing agent (Halifax, UK). Each sample underwent individual washing without acidification.

The test of sensory properties was conducted in accordance with the PN-EN ISO 6529:2005 standard [24] to visualise the influence of chemical stimulus on changes in conductive properties in printed and embroidered samples. Its purpose was to determine the resistance of materials with potential usage as protective clothing to the penetration of gases and liquids.

An acidic and alkaline perspiration resistance test was conducted in line with the PN-EN ISO 105-E04 standard “Textiles-Colour fastness tests-Part E04: Colour fastness to sweat” [25]. However, the study was performed to check the changes in surface resistance before and after contact with chemical solutions constituting acidic and alkaline sweat.

The prepared alkaline solution contained the following per litter:

- 0.5 g of L-histidine hydrochloride monohydrate (C_6_H_9_O_2_N_3_ × HCl × H_2_O);
- 5 g of sodium chloride (NaCl).

The solution was adjusted to pH 8.0 (0.2) with 0.1 mol/L sodium hydroxide solution.

An acidic solution was prepared containing the following per litter:

- 0.5 g of L-histidine hydrochloride monohydrate (C_6_H_9_O_2_N_3_ × HCl × H_2_O);
- 5 g of sodium chloride (NaCl).
- 2.2 g of dihydrogen phosphate 2-hydrate (NaH_2_PO_4_ × H_2_O).

The solution was adjusted to pH 5.5 (±0.2) with 0.1 mol/L sodium hydroxide solution.

On all the samples before all the tests and before each test, surface resistance measurements were taken using Keithley 2000 digital multimeter in accordance (Solon, OH, USA) with the PN-EN 1149-1 standard [26].

The works selected organic and inorganic substances, i.e., two types of chemical compounds that differ in composition and physicochemical properties. Organic substances are covalent compounds containing carbon, occurring in living organisms and their derivatives, while inorganic substances are ionic or covalent compounds that do not contain carbon and occur in nature. Both types of substances have different applications in industry and everyday life. Therefore, the authors selected in this article studies were related to apolar organic solvents-toluene, polar protic-methanol and polar aprotic-acetone. The measuring station for measuring changes in surface resistance under the influence of the presence of gas and chemical substances consisted of a glass chamber, a thermocouple, and a thermohydrometer to maintain the temperature and air humidity inside the chamber at 23 °C and 25% relative air humidity, respectively, glass dome with electrodes connected to a Keithley multimeter, and a pump injecting gas from the chamber into the dome. Photos of the laboratory station are in the article written by Krucińska et al. [27].

The study began with the introduction of a liquid into the chamber, which, under the influence of the supplied heat, evaporated. After obtaining the appropriate gas concentration in the chamber, which was 100 ppm, the tested object was placed on the electrodes in our case, a textile base with modification. Then, it was covered with a dome. The multimeter was turned on. Readings were observed until the sample stabilised, and then the pump was turned on to supply gas to the dome while observing the changes in surface resistance. The sample was tested for approximately 60 s. The sample response was immediately visible. After the test was completed, the next test object had to be inserted, while ensuring that the gas concentration in the chamber was at the appropriate level.

## 3. Results

Table 2 presents the initial surface resistance values for all tested materials including printing and embroidery. The surface resistance of the parting was presented for illustrative purposes regarding how it can be different for individual materials having the same modification. Surface resistance serves as a parameter describing the sensor’s response to a chemical stimulus. Based on the resistance value, the sensory coefficient was calculated, which was discussed in the next part of the article.

The most favourable surface resistance values for printed samples were obtained in high surface mass cotton and polyester fabric. The highest resistance value was obtained in a material consisting of cotton and polyester with a flame-retardant finish–BV 170. Samples containing embroidery are characterised by appropriate surface resistance values to perform the analysis of planned application tests. The lowest values were achieved by cotton with polyester and flame-retardant finish, and it was 2.69 Ω, the highest by polyester with cotton blend and it was 9.27 Ω. After testing the behaviour of 15 materials on toxic gases, we were able to determine the best ones.

A surface resistance was tested after each of the tests but the visibility result will be presented as a sensory percentage response. This parameter was calculated for each sample and each test. Sensory resistance was calculated according to Formula (1).

(1) $$R_{rel}=\frac{R-R_{0}}{R_{0}}\cdot 100\%$$

where $R_{rel}$—Sensory percentage response, R—final resistance, and $R_{0}$—initial resistance.

Sensory sensitivity incidences were performed for 10 measurements for each tested variant and each tested gas, and then an average was taken, which is included in Table 3, Table 4, Table 5, Table 6, Table 7 and Table 8. Table 3 presents changes in the sensory percentage response for screen-printed samples, depending on the test conducted and under the influence of the presence of acetone. Changes in surface resistance are directly related to their sensitivity to changes in the environment associated with the appearance of gas vapours. This is due to the fact that the appearance of gases causes chemical changes in the structure of conductive materials caused by swelling of the substrate, and changes in chemical bonds by adding groups from hazardous vapours. Usually, in such a case, conductivity deteriorates. The authors of the article decided to present the changes in the form of a percentage change coefficient, because in this way they are easier to analyse in terms of reaction strength.

Materials such as cotton show high reactivity to acetone, especially before modifications, but it can change for the worse after utility tests, such as washing and acidic sweating. Para-aramid reactions are at the desired level for this type of test, but different values are visible, for example, for the sample after the acidic sweat resistance test. Samples marked BV 170, BG 9500, and BG 1003 show significant changes in reaction with exceptions, such as the reaction of samples after the washing resistance test. In the case of sample BV 170, the unfavourable environment was acidic sweat. In Table 4 there are sensory coefficients for tests on acetone samples with embroidery modification.

Materials such as cotton, polyester knit and high surface mass cotton show moderate responses, but they are weakened after the fastness to washing, acidic, and alkaline sweat tests. The samples designated as high surface cotton and law surface mass cotton show desirable responses, because the sensor response is at a high level after each test. The sample with the working name performance has a good response before modifications, after the fastness to washing and after contact with alkaline sweat, but there is a visible deterioration in the sensory coefficient for the sample subject to the fastness to acidic sweat test; this parameter is only 6%. In Table 5, there are sensory percentage responses for tests on methanol samples with screen printing modification.

For samples with print and methanol, two materials can be distinguished, which, after each test, show a significant sensory coefficient; these are polyamide and linen. These results were at least 20%, and the highest result was as much as 98%. For some materials, only one result is significant in light of later sensory applications. For polyester, only the sample after washing has a particularly good sensory coefficient value, which is 91% with a negative decrease, while for polyester fabric the outstanding result is 67% with a negative decrease after testing the sample’s resistance to acidic sweat. Sample BV170 deserves a special mention, as the sample before the use tests has a parameter value of 85%, with a negative decrease. An analogous situation is for the sample with the research name Nomex after testing the resistance to alkaline sweat. In this case, the sensory coefficient was −65%. It is also possible to present samples that do not have a significant result for any test, an example of which would be low-mass cotton, BG 1003, or Performance. In Table 6, there are sensory coefficients for tests on methanol samples with embroidery modification.

In the case of sensors with embroidery and in response to methanol, there is no sample that would significantly stand out. The sensory coefficient value is significant in most cases and indicates high predispositions for the purpose of a gas sensor. The least expected results can be observed for samples with research names “Cotton” and “Performance”, which for two utility tests have sensory coefficient values below 30%. In Table 7, there are sensory coefficients for tests on toluene samples with screen printing modification.

In the case of the response of screen-printing samples to toluene, it is not possible to select the best material, considering all the conducted utility tests. Interesting results were obtained for high surface mass cotton, having a sensory percentage response of −75% for a sample not subjected to any utility tests, no conductivity after the washing resistance test, and after the acid sweat resistance test, the resistance values increased, and the tested parameter was 123%. Most materials not subjected to utility tests have responses no higher than 30%. In Table 8, there are sensory coefficients for tests on toluene samples with embroidery modification.

When analysing the reactions of embroidered materials to toluene, one can see a significant improvement in the response compared to screen-printed samples. Most samples have reactions above 50%. However, it is rare for a material to react similarly after all tests. Often, the direction of the reaction is different depending on the test carried out, meaning that during contact with toluene, the resistance increases or decreases depending on the tests carried out on the material. This applies to cotton, high surface mass cotton and polyamide. Acceptable results were obtained for para-aramid, polyester fabric and polyester knit, considering their high reactivity in all modifications.

Based on the selected method, the reactions were different when tested on the same gases. The presented data tables show that linen with print, low surface mass cotton with embroidery, and para-aramid with embroidery have the best response to toxic gases. After analysing the results, the system with the best predispositions for detecting gases was selected:

Acetone

- Para-aramid–embroidery–before testing;
- Law surface mass cotton–embroidery–before testing;
- Law surface mass cotton–embroidery–after washing resistance test;
- Law surface mass cotton–embroidery–after acid sweat resistance test;
- Law surface mass cotton–embroidery–after alkaline sweat resistance test

Methanol

- Linen–screen printing–before testing;
- Linen–screen printing–after washing resistance test;
- Para-aramid–embroidery–before testing.

Toluene

- Para-aramid–embroidery–before testing.

Below are graphics of selected sensors to visually present their response to selected gases. They have been aged in different configurations, considering the same material, modification, and gas vs. different utility studies, and one material, modification, before utility tests vs. different gases.

The design of the measuring station assumes that resistance measurements are performed on 5 cm wide electrodes spaced 1 cm apart. During the tests, it was not observed that the width of the samples had any significance for changes in resistance and, consequently, the sensory coefficient. The direction of gas flow in our device is circular, i.e., the gas flows from the bottom side of the sample into the measuring chamber and, from the other side, it flows back into the gas chamber. Therefore, we assume that the chamber is filled with gas during the test and the direction of gas flow is irrelevant. The samples react very quickly to changes in the environment, approx. 5–10 s, regardless of their width.

**Figure 2:**
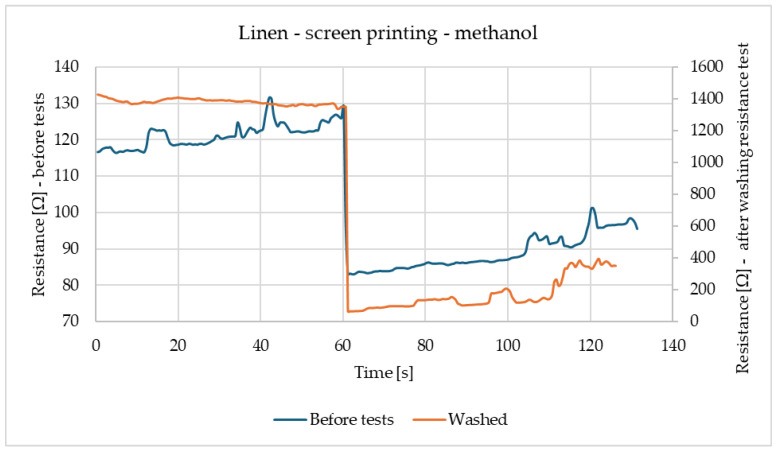
Results of Linen after screen printing with methanol.

Figure 2 shows the reactions of linen samples made using screen-printing methods before tests and after washing. Both samples have similar reactions, while being exposed to methanol. The only difference between samples is the scale of the reaction, while the sample before modifications has a reaction in the range of 80–130 Ω, the washed sample has higher resistance and reaction in the range of 100–1400 Ω. In both cases, resistance decreases after contacting the gas.

**Figure 3:**
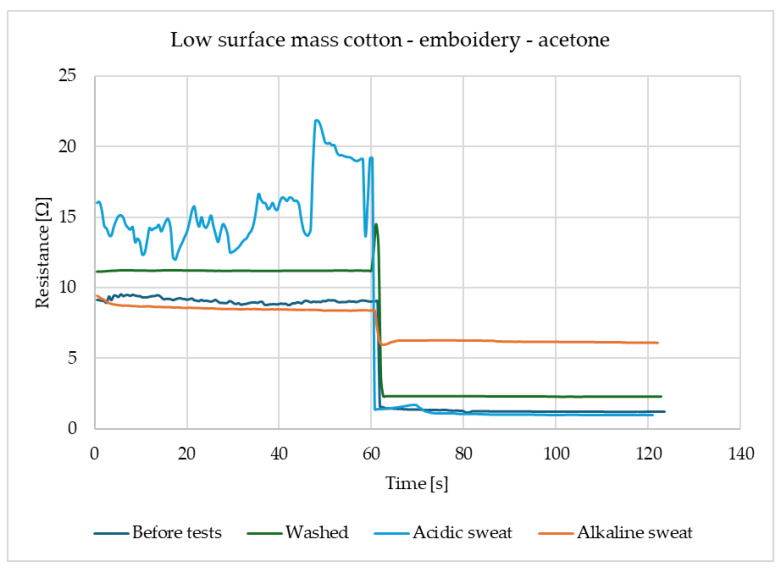
Results of law surface mass cotton after machine embroidery with acetone.

For the law surface mass cotton with the embroidery, presented in Figure 3, reactions to the acetone these reactions have a place for several types of samples and each of them has a similar scale. The response of the sensors is immediate and after contact with gas, the resistance drops significantly and stays on the same level. It shows that samples of law surface mass cotton with embroidery have responses at a proficient level after each of the utility tests.

**Figure 4:**
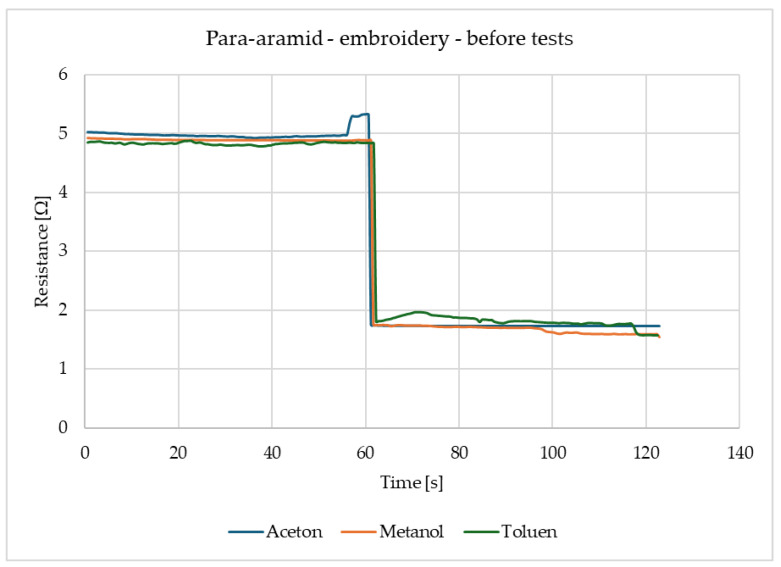
Results of para-aramid with embroidery before tests with acetone, methanol, and toluene.

Figure 4 shows that the surface resistance of para-aramid with embroidery decreases in the same way for all three gases. For acetone, methanol and toluene alike, the resistance immediately after gas detection decreases and remains at the same level. It should be emphasised that the readings of these samples are stable.

For all the above samples simulations were made. They consisted of vaporising the harmful gas alternately with ambient air. In everyday work, the gas sensor should not be disposable and must have the ability to detect gases multiple times. The graphs show the changes in surface resistance represented by the sensory percentage response. Sensory percentage responses were calculated based on Formula (1). For a clearer presentation of the results, the sensory percentage response has not been given in percentage terms. The name of the horizontal axis-exposure number means the interval in which the harmful gas or ambient air was present in the dome, for which the sensory percentage response was calculated. The names preceding the change in the direction of the graph mean the moment of introducing a given reagent into the dome. The sensory sensitivity graphs include sample results for the sensory sensitivity of the tested individual samples in subsequent vaporisation and cleaning cycles.

**Figure 5:**
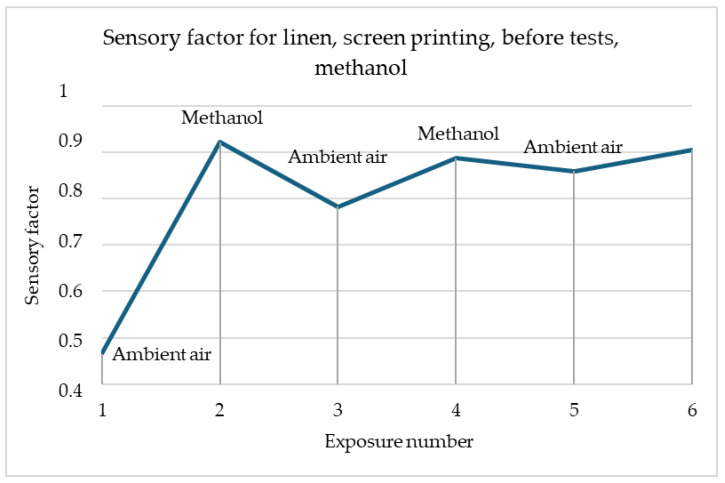
Sensory percentage responses change during ambient air and methanol exposition on linen after screen printing and before testing.

The graph presented in Figure 5 clearly shows the changes in the sensor response to the gas currently present in the dome. During exposure to methanol, a decrease in the sensory coefficient can be observed, while for air, there is an increase in value. This means that the surface resistance of the sample decreased in contact with methanol while improving the current flow. The reactions are repeatable, but with each subsequent exposure, they are not as strong. This may be due to the decreasing concentration of the harmful gas in the test chamber.

**Figure 6:**
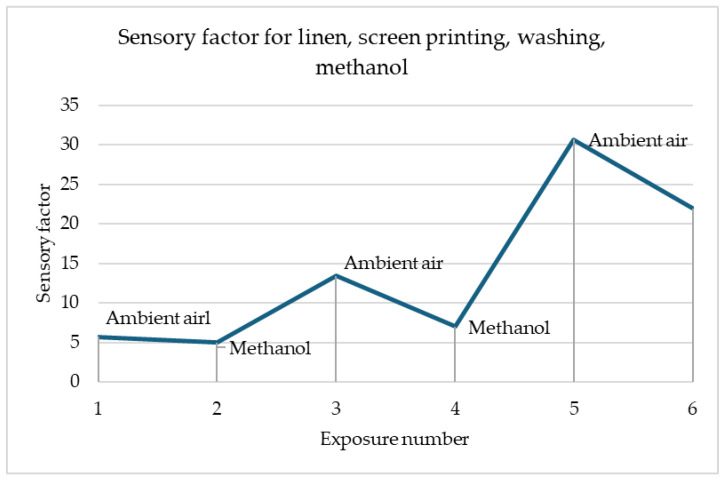
Sensory percentage response during ambient air and methanol exposition on linen after screen printing and washing.

Figure 6 shows the changes in the sensory percentage response for printed linen after the washing fastness test in response to contact with methanol. An inverse relationship can be seen here compared to the case of the sample before the utility tests. The parameter decreases due to contact with methanol and increases when exposed to air. The greatest changes in the sensory coefficient values are noticeable for the third exposure. This is a particularly good prognosis that the sensor can be used multiple times.

**Figure 7:**
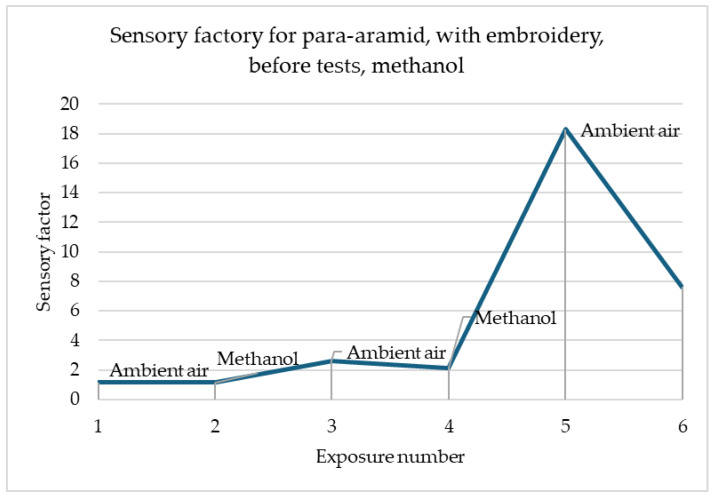
Sensory percentage responses change during ambient air and methanol exposition on para-aramid after embroidery and before modification.

In the simulation shown in Figure 7, the sensor’s reactions to the gas change are noticeable, with the third reaction to the presence of methanol being many times more intense than the first and second. There is a relationship that when exposed to methanol, the sensory coefficient value increases, and when exposed to air, it decreases. The increase and decrease are proportional to each other.

**Figure 8:**
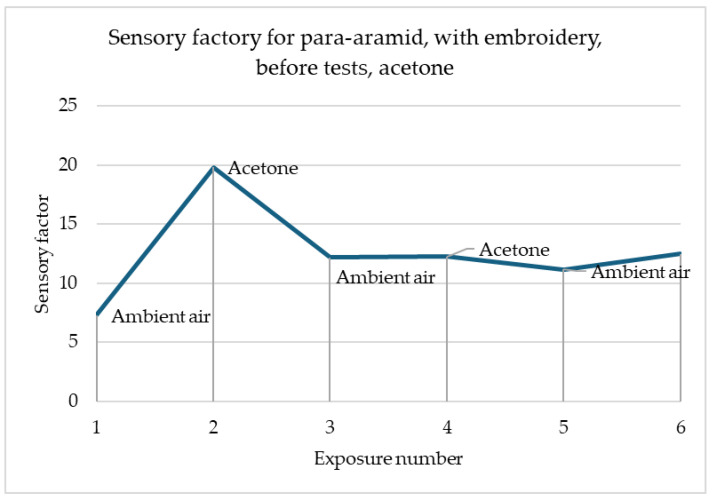
Sensory percentage responses change during ambient air and acetone exposition on para-aramid after embroidery and before modification.

In the simulation shown in Figure 8, noticeable reactions to the gas change were observed. An interesting phenomenon can be observed that the graph of the same sample for acetone is a mirror image of the graph for methanol in terms of the intensity of the reaction to subsequent exposures to harmful gas. Acetone causes a decrease in the sensory coefficient value, while air causes an increase. The first reaction is the strongest in comparison to the subsequent ones. It is likely that the sample had too little time to wash out the harmful gas molecules after first contact and no meaningful change was observed during the second exposure.

**Figure 9:**
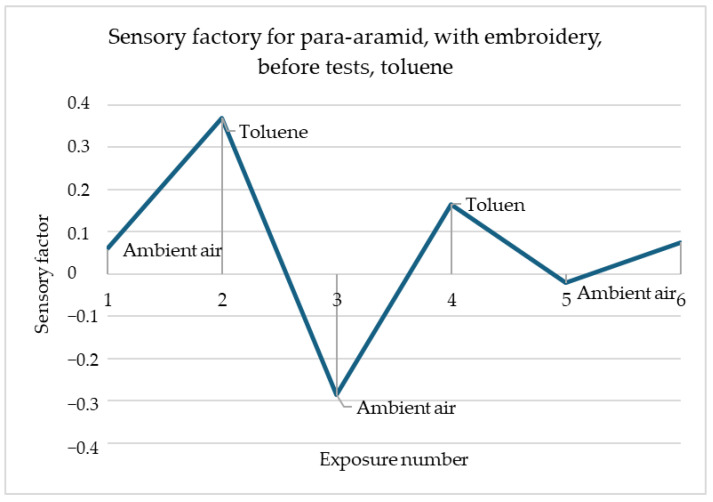
Sensory percentage responses change during ambient air and toluene exposition on para-aramid after embroidery and before modification.

In this simulation (Figure 9), the reactions were noticeable, but not in such a strong way as previous ones. The sensory percentage response was weaving between small values. The first reaction to toluene was the decreasing of resistance and the sensory percentage response. Ventilation caused a high increase in value. The second contact with the toluene caused a significant decrease in sensory percentage response value. Next, the ventilation caused another increase but not such strong as the previous one. However, the reactions at most points were noticeable and there were visible moments when there was a change in exposure to a given gas. In this case, it is important to test this material with another type of embroidery and check the significance of the response.

**Figure 10:**
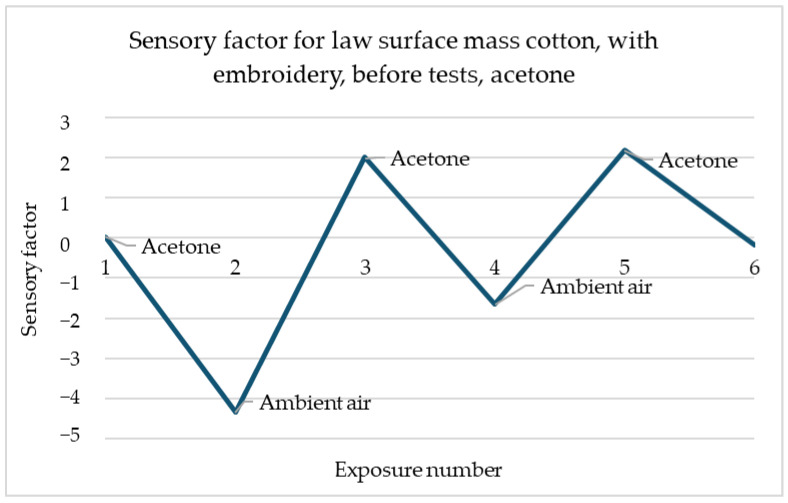
Sensory percentage responses change during ambient air and acetone exposition on law surface mass cotton after embroidery and before modification.

The simulation presented in Figure 10 presents a perfect reaction for every fabric contact with acetone. Every time, when it was supplied to the material sensory percentage response, it immediately decreased. Although acetone was fast evaporating, the response was sufficiently sustained to maintain the change in resistance throughout its influence. Importantly, changes in sensory percentage response values occurred always the same way—increases for ambient air and decreases for acetone, which facilitates the construction of a possible detection system.

**Figure 11:**
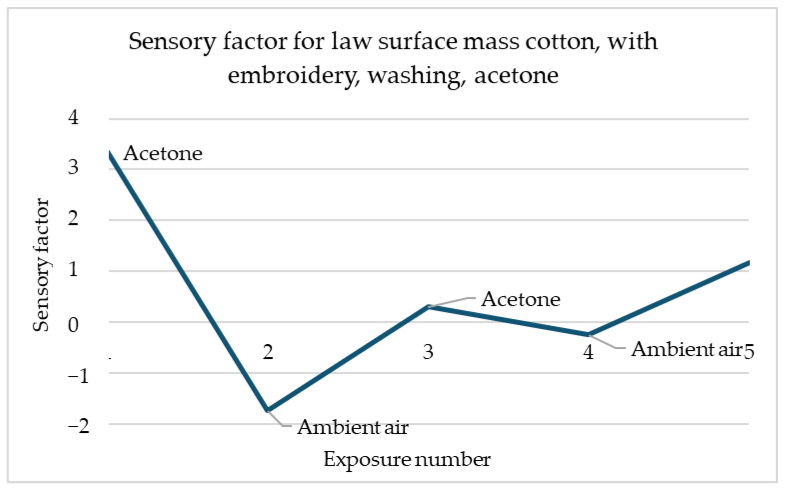
Sensory percentage responses change during ambient air and acetone exposition on law surface mass cotton after embroidery and washing.

Figure 11 shows the sensory response of cotton with low surface mass with embroidery in response to acetone after washing. The same direction of response to acetone is noticeable. The sensory percentage response decreases with each of the two exposures. The second response is slightly less significant than the first.

**Figure 12:**
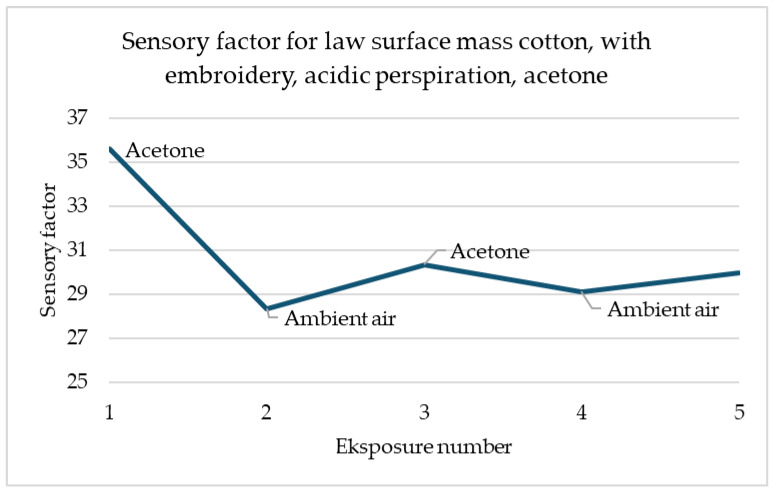
Sensory percentage responses change during ambient air and acetone exposition on law surface mass weave cotton after embroidery with acidic perspiration.

In the example shown in Figure 12, the relationship between the decrease in the sensory coefficient value and the increase in air is also visible. The first exposure is characterised by the highest decrease in value than each subsequent one. However, any change in gas in the dome is noticeable.

**Figure 13:**
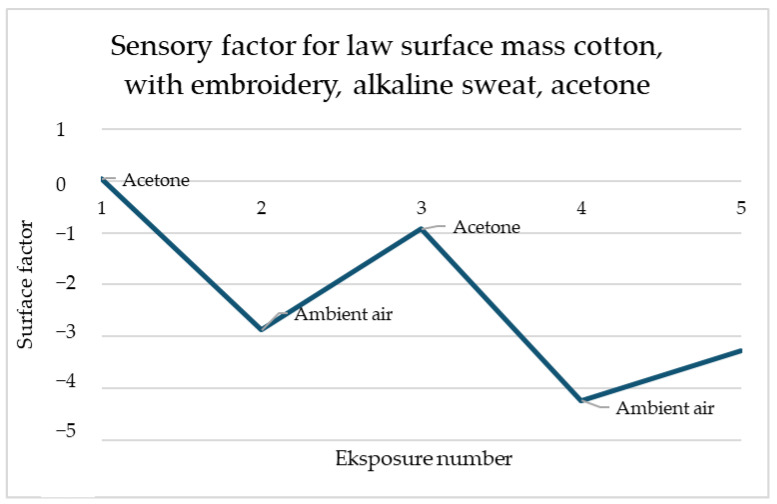
Sensory percentage response changes during ambient air and acetone exposition on law surface mass cotton after embroidery with alkaline perspiration.

The simulation for alkaline sweat (Figure 13) is more promising than for acidic (Figure 12). This graph shows the sample after the alkaline sweat resistance test for the sample with embroidery. A repeatable response is noticeable for each change in the environment. For both acetone contacts, the sensory coefficient at once decreased. The return to the original resistance with the ambient air may not be so spectacular, but it also appears momentarily and always increases. This observation is very optimistic for the easier possibility of designing a work detection system.

In Figure 5 and Figure 6, the greatest changes in the sensory coefficient values were observed during the third exposure. This type of behaviour may be caused by insufficient time for the sample to clear itself of gas fumes. There are cases where as in the case of the sample of printed linen fabric after washing processes, changes may have occurred in the structure of the fibres themselves, which, for example, as a result of damage, results in better interference into the depths of the material structure (the material swells faster, which improves sensitivity). A similar situation occurs in the case of para-aramid fabric after the embroidery process, in which the fibres may have been damaged. In most samples, the strongest reaction is observed after the first cycle of exposure to gas fumes. This is related to the fact that the sample has its first contact with a toxic substance. It is assumed that the sensors should be calibrated before first use so that they give the correct signal. In addition, each sensor must have time to expel the fumes that have entered it from its structures. Preliminary studies show that about 30 min is enough to permanently remove toxic substances from their interior before reuse. The authors have so far conducted a maximum of 30 attempts to repeat the correctness of operation on selected materials. It can be stated that the sensors worked correctly all the time. However, it should be remembered that when designing software for collecting information, the sensing coefficient should be assumed, not the resistance, because it can change after various processes.

## 4. Discussion

Fifteen materials with two modifications, screen printing and machine embroidery, were tested. Their ability to detect harmful gases such as acetone, methanol and toluene was tested. Samples that had not been subjected to utility tests were tested, as well as those after the washing resistance test and the acid and alkaline sweat resistance test. The surface resistance of the initial samples was tested to illustrate the change in this parameter after subsequent tests and to calculate the sensory coefficient based on the results obtained. The sensory coefficient is intended to clearly indicate by its value the percentage increase or decrease in surface resistance in response to contact of the detection system with a noxious gas. This coefficient had various values, showing that despite the same modification, the substrate plays a significant role in the case of detection properties.

Analysing the research results presented in Section 3, it can be observed that in the case of samples on which the embroidery was made, in most cases, the surface resistance improved for all gases. This is related to the fact that the conduction mechanism occurred on the surface of the yarns used for the embroidery process, without the participation of the textile substrate on which they were made. In cases where the conductivity deteriorated, it can be suspected that the yarns were damaged and their swelling occurred, which could cause the formation of energy holes. The fundamental role in the conduction mechanism of electrically conductive polymers is played by the strong interaction of the charge carrier with the nearest atoms of the chain on which the carrier is located. This is accompanied by local changes in the chemical structure of the chain. The concept of quasiparticles can be used to describe the mechanism of such interaction. The transport of the charge carrier is a series of oxidation-reduction reactions. By doping, an undoped polymer can be changed from an insulator into a conductor. In the terminology of solid-state physics, oxidation and reduction processes correspond to p-type and n-type doping, respectively. During p-type doping, an electron is extracted from the highest occupied molecular orbital of the polymer to the dopant, creating a hole. Conversely, an electron is transferred from the doped species to the lowest unoccupied molecular orbital of the polymer in the case of n-type doping. The conduction mechanism of polymers, i.e., the creation of free charge carriers and their transport, is possible thanks to conjugated double bonds. In the case of samples printed in addition to the print from carbon structures on the surface of textile materials, the substrate itself could have an influence on the changes in conductivity, which could swell under the influence of gas vapours. This was most visible for methanol vapours. The measurement signal generated in resistive gas sensors results from changes in the electrical conductivity of the receptor–transducer element, which occur under the influence of the tested substances. Chemisorption occurring on the surface of a chemically sensitive material often leads to the transfer of electric charge between the semiconductor and the tested substance. Electron transfer between adsorbed molecules and a semiconductor means a change in the concentration of charge carriers, and thus a change in the electrical conductivity of the receptor–transducer element. A very important role is played by the charges and electric field generated in the near-surface region of the semiconductor crystal. This is justified by the solid-state theory. It states that the wave functions of electrons located at the interface decay exponentially with distance from the semiconductor surface, which is why these carriers cannot move towards the interior of the crystal. This means that electrons accumulate primarily near the surface of the solid, where there are no restrictions on changing their position. Therefore, they can participate in processes that enable the generation of a measurement signal. In many electronic phenomena occurring in chemically sensitive resistors, surface states are of great importance. From a physical point of view, these are energy levels generated in the band gap of the semiconductor. Their concentration, energy position, and cross-section for charge carrier capture depend on, among other things, the properties of the chemically sensitive material, the state of its surface, and the characteristics of the adsorbed gases. Surface states occurring in semiconductor crystals used in resistive sensors can be spontaneous or non-spontaneous. The first group arises because of broken bonds, as well as chemical and structural defects. Unsaturated bonds are particularly important for the operation of resistive sensors.

Depending on the gas, substrate, modification and stage of testing, different behaviours of the sensory coefficient values were observed. In some cases, these values increased, and for others, they decreased. Significant changes can be noticed in values as well as less noticeable ones. The degree of change was also dependent on the previously mentioned factors. For example, in the case of BG 1003 and BG 9500 materials, after screen printing and washing resistance testing, a sensory index of 0% was recorded. This means no change in surface resistance during exposure to the harmful gas. For the linen sample with embroidery, a sensory index value of 174% was recorded in response to methanol. In contrast, for the positive value for the BV 170 sample with embroidery, a value of −100% was recorded in response to acetone, which means that the surface resistance values decreased during exposure to harmful gas.

The general relationship for all tested materials is a greater number of responses in the case of samples after modification with embroidery than after screen printing. The percentage of effectiveness of the screen-printing method is 17.22%, and in the case of embroidery, it is 75%. In terms of durability of modifications, machine embroidery also comes out on top here. Many more readings were obtained for embroidered samples for each test variant than for screen printing. Screen printing is great for making large-format sensors, but for smaller shapes, machine embroidery is better. The first of the tested materials, provisionally called Cotton after embroidery in the case of acetone, no significant changes could be noted for alkaline sweat, when exposed to methanol, samples after resistance to washing and to acidic sweat reacted visibly; in the case of toluene, only the lack of a significant reading was for acidic sweat. For the screen-printed samples, the desired response was obtained for the samples before the acetone tests and after the methanol washing resistance test. In the case of polyamide, for samples after machine embroidery, we can distinguish those after the washing resistance test and alkaline sweat for acetone, before and after the tests for acidic and alkaline sweat for methanol, and after washing resistance, as well as acidic and alkaline sweat for toluene. In the case of samples after screen printing modification, we can distinguish those after the washing resistance test and acidic sweat for methanol, and after the washing resistance test for toluene. For polyester knitwear, a significant sensory coefficient can be seen for the sample after resistance to washing in the presence of methanol. It also noted the expected results for toluene, in this case, the only sample that did not achieve the expected result, was the one before all tests. For samples with metal thread embroidery, toluene should be distinguished again. For this gas, all variants had the desired values of the sensory coefficient. In addition, it is worth emphasising the samples for methanol, only the sensor after the washing resistance test did not have a significant result. Significant responses were also given by samples after resistance to washing and to alkaline sweat for acetone. High surface mass cotton had meaningful results for the acid sweat resistance sample for acetone, for the post-wash fastness sample for methanol, and for all but the pre-test sample for toluene. The embroidery samples responded excellently to toluene, and for acetone, the pre-test sample was missing, and for methanol, the post-wash fastness sample was missing. Unfortunately, the material called law surface mass cotton did not react to any gas after modification with screen printing. However, after modification with embroidery, it worked 100% in the case of toluene and methanol, and in the case of acetone only the sample after the test of resistance to alkaline sweat was missing. Linen had the most readings on samples after screen printing modification. A significant result was noted for acetone for the sample before the tests, as many as three readings were noted for methanol, for samples after the washing and sweat tests, and two samples reacted to toluene, after the washing and alkaline sweat resistance tests. Samples after embroidery modification noted an excellent result for methanol, all system variants had a significant sensory coefficient. For toluene, there was no significant result only for the sample after the washing resistance test, and in the case of acetone, two samples were noted, after the acid and alkaline sweat resistance test. Para-aramid after screen printing modification reacted to acetone in the case of the sample before the tests and after the washing resistance test, to methanol in the case of the sample after the alkaline sweat resistance test, and to toluene in the case of the sample after the washing resistance test. The embroidered material coped very well with methanol and for all variants had a high sensory index, for acetone only the sample before the tests did not have a significant result, and for toluene the sample after the alkaline sweat resistance test was missing. The viscose fabric in the case of screen printing did not react to any gas for any variant of the samples. In the case of machine embroidery, the samples reacted to acetone after the sweat resistance test, to methanol the samples after the washing and sweat resistance test, and to toluene both before and after the washing resistance test. For the screen-printed modified polyester fabric, one significant reading was recorded for the sample after the acidic sweat resistance test in response to methanol. In the case of the second modification, only a significant response was missing for the samples after the alkaline sweat resistance test in response to acetone. The detection of methanol and toluene was passed with 100% success. The polyester with cotton blend fabric with the printed paste did not record any significant reading for acetone; for methanol, the detection properties could be assigned to the sample after the alkaline sweat resistance test, and for toluene, to the samples before tests and after the wash resistance test. This material was the only one after the embroidery modification that did not record any response also for acetone; however, the efficiency for methanol and toluene was 100%. BG9500 also recorded one significant result after screen printing modification for the sample after the acid perspiration test in response to methanol. In contrast, their embroidered samples mostly had a high sensory index. Acetone had significant responses for samples before and after the wash fastness test, methanol for samples after the wash fastness and perspiration test, and toluene for samples before and after the wash fastness test. BG1003 is another interesting case. After screen printing modification, no significant sensory coefficient result was recorded; however, for embroidered samples, no such result was recorded only for one sample. This was the case with the toluene response for the sample after the acid sweat resistance test. The BV170 material had three readings for printed samples. The samples after the acidic sweat resistance test reacted to acetone, and the samples before and after the alkaline sweat resistance test reacted to methanol. Unfortunately, no meaningful results were obtained for toluene. The opposite was the case with embroidered samples, for which the desired sensory coefficient was not obtained only for the sample after the alkaline sweat resistance test in response to toluene. Nomex only in the case of the sample after the alkaline sweat test recorded a significant result in response to methanol. For the embroidered samples, it had the smallest number of responses to gases. Only the sample after the alkaline sweat test reacted to acetone. The sample after the alkaline sweat test did not react to methanol and, in the case of toluene, the sample after the acidic sweat test did not react either. The performance material was another one that did not record any significant sensory coefficient value for printed samples. However, it was 100% correct in detecting toluene for embroidered samples. Two samples reacted to methanol after sweat resistance tests, and to acetone after washing and alkaline sweat tests. All the exposures performed had to be analysed in detail and those textronics sensors that achieved the most promising results had to be selected.

It was law surface mass cotton knitwear that reacted to ethanol, after modification with embroidery, before and after all use tests, para-aramid knitwear, after modification with embroidery before use tests, reacted to all gases and linen knitwear, after screen printing for methanol, as well as before test and after washing test. Considering the simulation results, repeated reactions were observed in the presence of gases. It was observed that, after contact with harmful gases, surface resistance increases or decreases for every exposure. Graphs of materials in different configurations are presented. Most importantly, the surface resistance after the first contact with a harmful gas does not remain at the same level, but after contact with air, it returns to its initial or similar initial level. It is also worth emphasising the short intervals of exposure to harmful gas. In real conditions, the sensor would have incomparably more time to cleanse itself of harmful gas molecules, thanks to air, and have contact with the highest value of the concentration of the substance. In the case of a laboratory test, it is possible that the textronic sensor contained a small percentage of gas molecules from the earlier exposure with each subsequent exposure. Therefore, each subsequent change in the surface resistance value may not have been as visible as the initial one. A concentration of 100 ppm was also used to conduct the tests in a safe manner.

These studies show the possibility of creating textronic systems sensitive to harmful gases, resistant to wear and not losing their detection properties during everyday use factors and sensors of the “all in one” type, i.e., reacting not only to one gas but also for many simultaneously.

## 5. Conclusions

The results obtained allowed us to select materials that respond best to the gases selected for the above article. Samples were modified in two ways to determine which method is more efficient in terms of operation, resistance level and detection. Despite the use of the same paste and metal thread components, textile sensors exhibit different surface resistance values. The base material significantly affects the conductive properties of the resulting textronic systems. The embroidered samples performed better, achieving higher sensitivity than those after screen printing. The samples modified by screen printing, after testing, especially after the washing resistance test, in most cases significantly lost their conductivity. The reason for this is the breaking of bonds between the base material and the ink paste with carbon structures. During gas detection, the resistance of the samples decreased or increased, depending on the direction of the reaction; no correlation was observed between the method, the modification or the detected gas. The systems that have the most expected results are in the case of acetone: para-aramid-embroidery-before performance tests, fine cotton weave-embroidery-before performance tests, fine cotton weave-embroidery-after the washing resistance test, fine cotton weave-embroidery-after the acid sweat resistance test, and fine cotton weave-embroidery-after the alkaline sweat resistance test. The reason for selecting these samples is that they provided us with the best responses and responded by reducing or increasing the resistance significantly. However, to obtain an effective product, the tests must be extended to check how the materials will react under different environmental conditions, such as changing temperatures, humidity levels, pressures or the presence of other contaminants. In addition, it is important to extend the studies described to investigate other materials that could also prove to be effective sensors. Expanding the range of materials will enable us to identify potential alternatives that may be as or even more effective than those currently used. The greatest emphasis should be placed on conducting studies that will identify the best modification method and the best material that will respond in the same way under all conditions, regardless of pH level or whether; for example, the textronic system has been washed. Such extended studies will not only help to select the optimal materials for our sensor applications but also increase our knowledge of how materials respond to different environmental stimuli. This in turn will contribute to the development of more advanced and versatile sensor solutions. Another avenue for development is to conduct studies on other harmful gases such as propane-butane or carbon monoxide, which are responsible for the highest number of poisonings. However, this requires a safe test facility that will not expose the tester to poisoning.

There is already some work on the ecological approach to textronic systems and research is underway on how to produce them to extend their life cycle and thus produce less waste [28]. In our times, caring for the environment is an important aspect and when introducing modern solutions, we should also take care of this area of the product cycle. Referring to the ecological aspect, the use of the textronic systems described in the article when used in home products such as curtains or blinds fit into this canon. During the year, such products are washed about two times and are not exposed to acidic and alkaline sweat. A long life is expected for this type of solution. However, if the time comes to dispose of such a product, under no circumstances can it be thrown into a commonly used garbage bin. Textronic waste is treated as e-waste. These products can be recycled. The first way is using thermal pyrolysis [29]. These aspects are still being intensively analysed, and research is ongoing on how to best use textronic waste.

The direction of development for the tested systems is their stabilisation and strengthening of the repeatability of results. Additionally, the systems should be expanded in terms of an alarm system that will visually or audibly inform people in its vicinity about the danger. The modernization of textronic systems reacting to harmful gases is the creation of sensors that do not need a source of energy or are integrated with sources of energy by nature, such as solar panels.

In preliminary studies, detection tests were conducted from 50 ppm, unfortunately, the response was very low; therefore, the article presents tests for 100 ppm, which constitute a very good response. Considering the fact that the human nose detects much higher concentrations, and sometimes a headache appears first, detection at the level of 100 ppm provides a wide range of applications. The reaction time to the appearing vapours did not exceed 5 s. Therefore, the authors considered that the sensor reacted very well. The authors also observed that increasing concentrations caused increasingly stronger reactions of percentage changes in the sensor. Preliminary tests were conducted in the range from 50 to 500 ppm.

All fifteen materials were tested with two modifications-embroidery and printing, to obtain information on what applications they can be intended for. As a result of our research, it is possible to propose an application for cotton clothing, which could have an acetone sensor placed. A methanol sensor for home use could be placed on linen curtains or drapes. In turn, the sensor for various harmful gases—in our case, acetone, methanol, and toluene—could be used in special firefighter uniforms made of Kevlar–para-aramid.

## Figures and Tables

**Table 1:** Research textiles and their characteristics.

Composition	Type	Name	Surface Mass [g/m^2^]
Cotton, elastane	Knitwear	Cotton	255
Polyamide, elastane	Knitwear	Polyamide	224
Polyester, elastane	Knitwear	Polyester	116
Cotton (high surface mass)	Knitwear	Cotton	271
Cotton (low surface mass)	Knitwear	Cotton	103
Linen with a cotton blend	Knitwear	Linen	115
Para-aramid	Knitwear	Para-aramid	184
Viscose	Fabric	Viscose	118
Polyester	Fabric	Polyester	126
Polyester with a cotton blend	Fabric	Polyester/cotton	169
Cotton, Polyester, Static control	Knitwear	BG 9500	249
Cotton, Polyester, flame retardant finish	Knitwear	BG 1003	360
Nomex, Para-aramid, Antistatic fibre P140	Fabric	BV 170	159
Nomex, Para-aramid	Fabric	Nomex	236
Nomex, Para-aramid	Fabric	Performance	195

**Table 2:** Initial surface resistance of the tested textural systems.

Screen Printing		
Materials	Surface Resistance [Ω]	Standard Deviation [Ω]
Cotton	1786.40	105.45
Polyamide	11,934.78	997.75
Polyester knitwear	357.50	14.24
High surface mass cotton	59.96	3.53
Law surface mass cotton	132.26	2.26
Linen	113.62	8.86
Para-aramid	564.93	21.63
Viscose	19,634	9.76
Polyester fabric	91.29	4.23
Polyester with a cotton blend	133.49	9.81
BG 9500	2011.10	69.03
BG 1003	388.77	11.12
BV 170	44,283.17	769.52
Nomex	367.06	19.90
Performance	39,957.54	1547.06
**Embroidery**		
**Materials**	**Surface Resistance [Ω]**	**Standard Deviation [Ω]**
Cotton	4.35	0.16
Polyamide	3.49	0.25
Polyester knitwear	2.95	0.68
High surface mass cotton	4.41	0.19
Law surface mass cotton	8.65	0.45
Linen	5.10	0.17
Para-aramid	3.62	0.23
Viscose	8.17	0.70
Polyester fabric	8.28	0.11
Polyester with a cotton blend	9.27	0.20
BG 9500	3.04	0.07
BG 1003	5.10	0.25
BV 170	2.58	0.24
Nomex	3.44	0.20
Performance	2.69	0.12

**Table 3:** Sensory percentage responses for tests on acetone on screen printed samples.

Acetone–Screen Printing				
Materials	Before Modifications	Washed	Acidic Perspiration	Alkaline Perspiration
Cotton	−69%	−6%	−6%	−1%
Polyamide	−8%	−10%	−3%	−5%
Polyester knitwear	29%	−7%	−1%	5%
High surface mass cotton	−1%	−13%	50%	2%
Law surface mass cotton	−34%	−13%	−7%	−6%
Linen	−47%	−14%	−6%	5%
Para-aramid	75%	47%	−21%	−7%
Viscose	6%	14%	−7%	−3%
Polyester fabric	−13%	−20%	2%	−5%
Polyester with a cotton blend	−13%	−9%	14%	−15%
BG 9500	−8%	0%	−32%	−13%
BG 1003	−43%	0%	−34%	−13%
BV 170	−17%	14%	−55%	−2%
Nomex	4%	3%	−6%	1%
Performance	−14%	−3%	14%	−5%

**Table 4:** Sensory percentage responses for tests on acetone on embroidered samples.

Acetone–Embroidery				
Materials	Before Modifications	Washed	Acidic Perspiration	Alkaline Perspiration
Cotton	−50%	−40%	−84%	−10%
Polyamide	15%	−81%	−3%	−69%
Polyester knitwear	32%	−92%	−34%	−85%
High surface mass cotton	−30%	−52%	−58%	−54%
Law surface mass cotton	−87%	−79%	−94%	−27%
Linen	16%	−28%	−52%	−84%
Para-aramid	−8%	−67%	−53%	−71%
Viscose	2%	−28%	−63%	−81%
Polyester fabric	−84%	−41%	−97%	14%
Polyester with a cotton blend	−23%	−17%	−12%	5%
BG 9500	−64%	−66%	−21%	−28%
BG 1003	−92%	−98%	−96%	−89%
BV 170	−44%	−81%	−86%	−100%
Nomex	5%	−24%	−29%	−60%
Performance	−32%	−74%	6%	−77%

**Table 5:** Sensory percentage responses for tests on methanol on screen printed samples.

Methanol–Screen Printing				
Materials	Before Modifications	Washed	Acidic Perspiration	Alkaline Perspiration
Cotton	15%	−40%	−20%	2%
Polyamide	−32%	78%	98%	−20%
Polyester knitwear	−5%	−91%	−17%	−22%
High surface mass cotton	8%	18%	−26%	−2%
Law surface mass cotton	5%	−18%	17%	−4%
Linen	−26%	−74%	−73%	55%
Para-aramid	26%	−21%	−1%	−58%
Viscose	2%	−21%	3%	12%
Polyester fabric	24%	31%	−67%	−5%
Polyester with a cotton blend	2%	2%	−7%	−87%
BG 9500	−1%	13%	−52%	−23%
BG 1003	9%	3%	−33%	1%
BV 170	−85%	7%	−30%	50%
Nomex	15%	−5%	4%	−65%
Performance	28%	−11%	−4%	0%

**Table 6:** Sensory percentage responses for tests on methanol on embroidered samples.

Methanol–Embroidery				
Materials	Before Modifications	Washed	Acidic Perspiration	Alkaline Perspiration
Cotton	14%	−64%	−57%	−27%
Polyamide	188%	−6%	−98%	−84%
Polyester knitwear	−55%	−26%	−73%	−49%
High surface mass cotton	−75%	−24%	−81%	84%
Law surface mass cotton	−69%	−42%	−78%	197%
Linen	−95%	174%	−81%	−66%
Para-aramid	−67%	−68%	−79%	−54%
Viscose	−29%	−71%	−59%	−94%
Polyester fabric	−51%	−56%	−59%	−88%
Polyester with a cotton blend	−92%	−86%	−70%	−85%
BG 9500	−32%	−84%	−96%	−71%
BG 1003	−82%	−57%	−95%	−59%
BV 170	66%	−70%	−82%	−74%
Nomex	−81%	−42%	−91%	22%
Performance	−17%	−16%	−77%	−87%

**Table 7:** Sensory percentage responses for tests on toluene on screen printed samples.

Toluene–Screen Printing				
Materials	Before Modifications	Washed	Acidic Perspiration	Alkaline Perspiration
Cotton	−33%	4%	3%	0%
Polyamide	−32%	80%	−6%	−1%
Polyester knitwear	−1%	−85%	−90%	−57%
High surface mass cotton	−75%	0%	123%	2%
Law surface mass cotton	6%	4%	10%	2%
Linen	−7%	168%	−14%	−73%
Para-aramid	−21%	144%	−7%	−19%
Viscose	13%	−6%	−2%	23%
Polyester fabric	2%	8%	6%	−12%
Polyester with a cotton blend	−79%	−61%	−29%	0%
BG 9500	−15%	0%	−10%	−8%
BG 1003	3%	8%	39%	−22%
BV 170	33%	10%	0%	−3%
Nomex	−22%	−10%	1%	136%
Performance	2%	3%	−4%	4%

**Table 8:** Sensory percentage responses for tests on toluene on embroidered samples.

Toluene–Embroidery				
Materials	Before Modifications	Washed	Acidic Perspiration	Alkaline Perspiration
Cotton	96%	−93%	−12%	−75%
Polyamide	13%	−93%	−88%	−84%
Polyester knitwear	−92%	−66%	−88%	−41%
High surface mass cotton	−89%	88%	−83%	−92%
Law surface mass cotton	−77%	1%	34%	−71%
Linen	−68%	−12%	−98%	−93%
Para-aramid	−64%	−89%	−84%	−38%
Viscose	−83%	−81%	−10%	−70%
Polyester fabric	−86%	−47%	−90%	−75%
Polyester with a cotton blend	−98%	−55%	−87%	−79%
BG 9500	−81%	−57%	−2%	−37%
BG 1003	−55%	−58%	8%	−97%
BV 170	−89%	−90%	−74%	17%
Nomex	−91%	−53%	14%	−100%
Performance	−38%	−77%	−86%	−93%

## Data Availability

The original contributions presented in this study are included in the article. Further inquiries can be directed to the corresponding author.
